# An automatic approach for constructing a knowledge base of symptoms in Chinese

**DOI:** 10.1186/s13326-017-0145-x

**Published:** 2017-09-20

**Authors:** Tong Ruan, Mengjie Wang, Jian Sun, Ting Wang, Lu Zeng, Yichao Yin, Ju Gao

**Affiliations:** 1 East China University of Science and Technology, Shanghai, China; 2 Shanghai Shuguang Hospital, Shanghai, 200025 China

**Keywords:** Knowledge base, Symptoms in Chinese, Linked data, Information extraction

## Abstract

**Background:**

While a large number of well-known knowledge bases (KBs) in life science have been published as Linked Open Data, there are few KBs in Chinese. However, KBs in Chinese are necessary when we want to automatically process and analyze electronic medical records (EMRs) in Chinese. Of all, the symptom KB in Chinese is the most seriously in need, since symptoms are the starting point of clinical diagnosis.

**Results:**

We publish a public KB of symptoms in Chinese, including symptoms, departments, diseases, medicines, and examinations as well as relations between symptoms and the above related entities. To the best of our knowledge, there is no such KB focusing on symptoms in Chinese, and the KB is an important supplement to existing medical resources. Our KB is constructed by fusing data automatically extracted from eight mainstream healthcare websites, three Chinese encyclopedia sites, and symptoms extracted from a larger number of EMRs as supplements.

**Methods:**

Firstly, we design data schema manually by reference to the Unified Medical Language System (UMLS). Secondly, we extract entities from eight mainstream healthcare websites, which are fed as seeds to train a multi-class classifier and classify entities from encyclopedia sites and train a Conditional Random Field (CRF) model to extract symptoms from EMRs. Thirdly, we fuse data to solve the large-scale duplication between different data sources according to entity type alignment, entity mapping, and attribute mapping. Finally, we link our KB to UMLS to investigate similarities and differences between symptoms in Chinese and English.

**Conclusions:**

As a result, the KB has more than 26,000 distinct symptoms in Chinese including 3968 symptoms in traditional Chinese medicine and 1029 synonym pairs for symptoms. The KB also includes concepts such as diseases and medicines as well as relations between symptoms and the above related entities. We also link our KB to the Unified Medical Language System and analyze the differences between symptoms in the two KBs. We released the KB as Linked Open Data and a demo at https://datahub.io/dataset/symptoms-in-chinese.

## Background

Medical knowledge bases (KBs) play an important role in healthcare research. Existing KBs vary from coding systems such as ICD10 [1], terminology systems such as UMLS [2], clinical ontology systems such as SNOMED CT [3] to medical databases such as DrugBank [4]. The major objectives for these KBs are to provide knowledge to medical workers and to promote standardization and interoperability for biomedical information systems and services. Besides, there exist many different types of biomedical KBs. For example, SIDER [5], and AMDD [6] contain drug-related information. Diseasome [7], ParkDB [8], and ChemProt [9] describe disease and disease-related gene information. These KBs are necessary in automatically processing and analyzing electronic medical records (EMRs) and then form the basis of the upper information applications such as clinical decision support systems.

Currently there are many general-purpose KBs built by automatic approaches. The DBpedia project [10] extracted structured information from Wikipedia and published them on the Web. YAGO [11] was derived from Wikipedia, WordNet, and GeoNames. NELL [12], SOFIE [13], and PROSPERA [14] extracted data from the Web. The input data for NELL consisted of an initial ontology as well as a small number of instances. SOFIE extracted ontological facts from natural language texts and linked the facts into an ontology. PROSPERA relied on the iterative harvesting of n-gram-itemset patterns to generalize natural language patterns found in texts.

There are also some studies in the medical field which construct KBs automatically. Ayvaz et al. [15] built a dataset of drug-drug interaction information from existing datasets including DrugBank, KEGG, NDF-RT, and so on. Ernst et al. [16] constructed a knowledge graph for biomedical science which extracted and fused data from scientific publications, encyclopedic healthcare portals and online communities. They used distant supervision in the extraction step, and used logical reasoning for consistency checking.

However, most KBs are in English. The KBs in Chinese are necessary in order to process the large amount of EMRs in Chinese, which has been accumulated since the wide adoption of hospital information systems a decade ago. Of all KBs, the symptom KB in Chinese is mostly required, since symptoms are the starting point of clinical diagnosis and reflect the evolution of diseases. We focus on symptoms and symptom-related entity extraction. Data sources and methods in the construction of our KB are different from previous work, and the experiments show that our KB gains roughly a higher precision than similar results in [16]. Our KB is constructed by fusing data automatically extracted from eight mainstream healthcare websites, three Chinese encyclopedia sites, and symptoms extracted from a lager number of EMRs as supplement. This automatic approach not only avoids a large amount of manual work, but also keeps up with changes when new entities and relations appear.

## Methods

### Data schema

The schema of our KB can be regarded as a simplified version of UMLS. UMLS contains complex taxonomy including physical objects, events, or even intellectual products. Since our KB focuses on symptoms, we only choose parts of UMLS related to our work, such as, “*Finding*”, “*Sign* *or* *Symptom*”, and “*Disease* *or* *Syndrome*”.

We have summarized five concepts for our KB driven by the requirements of processing and analyzing EMRs. Besides Symptom, we add four concepts directly related to symptom, namely, Disease, Medicine, Department, and Examination. Traditional Chinese medicine (TCM) describes symptoms that is different from Western medicine. For example, “Yin_deficiency” and“Qi_stagnation” are TCM symptoms which have no direct connection with Western medicine. In addition, TCM diagnosis and Western medicine diagnosis are two independent parts in EMR systems. TCM symptoms are included in the part of TCM diagnosis. Taking the above factors into consideration, Symptom is further divided into TCM Symptom and Symptom of Western Medicine, and Medicine is divided into TCM and Western Medicine similarly.

**Fig. 1:**
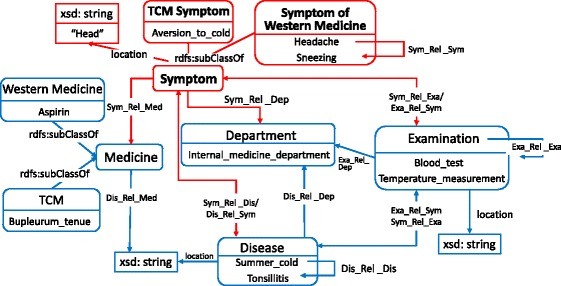
Schema Graph for Knowledge Base of Symptoms in Chinese. Each rectangle represents a concept and the bottom of each rectangle is an instance

The schema graph is shown in Fig. 1. The center of the graph is the concept Symptom. Symptom links to other concepts with relations such as *relevant_disease*, and has datatype properties such as *location*. The instances in Fig. 1 form a virtual scenario in clinical practice. A person has “Headache”, “Sneezing” and “Aversion_to_cold” which is a TCM symptom. Perhaps he goes to the “Internal_medicine_department”, and has the “Blood_test” and “Temperature_measurement”. Finally, he is diagnosed with “Summer_cold” complicating with Tonsillitis and is suggested to take some “Aspirin” and “Bupleurum_tenue”. Chinese usually take TCM and Western medicine together.

### Data extraction

**Fig. 2:**
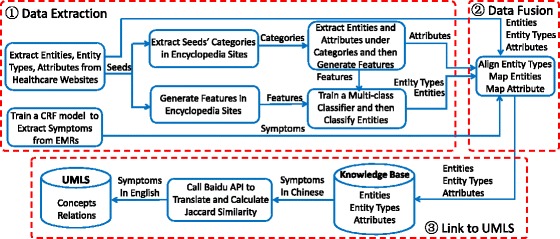
Workflow of Constructing the Knowledge Base of Symptoms in Chinese. It contains three steps: (*1*) Extract data from healthcare websites, encyclopedia sites and EMRs, respectively. (*2*) Align the extraction results. (*3*) Link our symptoms to concepts in UMLS

Figure 2 presents the overall workflow of constructing our KB. In the data extraction step, we first use specific HTML wrappers [17] to extract entities and attributes from semi-structured information in eight mainstream healthcare websites. Then, we extract entities from three Chinese encyclopedia sites. Entities obtained from healthcare websites are fed as seeds to train a multi-class classifier and classify entities from encyclopedia sites. Finally, we train a Conditional Random Field (CRF) model to extract symptoms from EMRs. The details are described below.

#### Data extraction from healthcare websites

We collect eight mainstream healthcare websites (See in Table 1). There are normally two kinds of web pages for each website. One is the list page containing a list of entities. The other is the detail page containing the detail description of a particular entity. All the above websites contain list pages of symptoms, diseases and medicines. However, list pages of department do not exist in *JIANKE*, *PCbaby*, or *fh*21, and list pages of examinations only appear in *JIANKE*, 120*ask*, and 39*Health*.

**Table 1:** Basic information of eight healthcare websites

Website name	URL
Familydoctor	http://www.familydoctor.com.cn/
JIANKE	http://www.jianke.com/
120ask	http://www.120ask.com/
QQYY	http://www.qqyy.com/
39Health	http://www.39.net/
99Health	http://www.99.com.cn/
Fh21	http://www.fh21.com.cn/
Pcbaby	http://www.pcbaby.com.cn/

We take the symptom extraction as an example. All the detail pages of symptoms in a website share similar layouts. There is a portion called “property box” in the detail page containing attribute-value pairs of an entity. We map the “property box” to properties in schema graph manually and extract attribute-value pairs with HTML wrappers. Since symptom may be a TCM Symptom or Symptom of Western Medicine, we use the “relevant department” attribute to classify. Specifically, if a symptom is related with a department containing “TCM” (e.g, Traditional Chinese Orthopedics, it will be labeled as TCM Symptom. The symptom will be tagged as Symptom of Western Medicine if it is related with a department without “TCM”. An entity can be labeled as both TCM Symptom and Symptom of Western Medicine. Finally, synonymous relations are crawled from the abstracts of entity pages with Hearst patterns described in [18]. For example, when applying a pattern “[entity1] is known as [entity2]” to sentence “hyperpyrexia is known as fever”, a “sameAs” relation between “hyperpyrexia” and “fever” is extracted.

We apply similar steps to other types of entities. We use heuristic information in the detail page of a medicine to distinguish TCM and Western medicine. If its description in detail page contains keywords such as “Chinese patent medicine” and “herbal medicine”, and it is Western medicine if its description contains “pharmaceuticals”, “chemicals”, and so on.

#### Data extraction from Chinese encyclopedia sites

We extract and classify entities from three largest Chinese encyclopedia sites (i.e. Baidu Baike, Hudong Baike, and Chinese Wikipedia).

Algorithm 1 shows the extraction process. First, we take entities from healthcare websites as seeds (denote as EntityList), and extract their categories in Chinese encyclopedia sites (denote as SeedCateSet).



Then we crawl entities belonging to SeedCateSet. Second, we classify SeedCateSet into low-confidence and high-confidence by calculating the ratio of seeds in these categories. Thirdly, for each entity in encyclopedia site, if its categories contain low-confidence categories, it will be regarded as noise and removed. We eventually collect 62,013 entities from Baidu Baike, 59,704 entities from Hudong Baike, and 2220 entities from Chinese Wikipedia.

In the classification step, we take entities extracted from healthcare websites as positive examples and entities from list pages of “health preservation”, “facial” and “psychology” in healthcare websites as negative examples. Then train a Decision Tree [19] classifier with seven labels: department, TCM, western medicine, symptom, disease, examination and other.

**Fig. 3:**
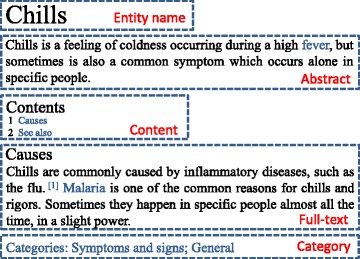
Five Fields of Entity Page in Encyclopedia Sites

The classifier uses two types of features, namely, word formation and word distribution. The features are obtained from five fields, namely, entity name, abstract, content, full-text, and category of entity page, as shown in Fig. 3 and Table 2. If an entity is classified as Symptom, we will use heuristic rules to further determine whether it is a TCM Symptom or Symptom of Western Medicine.

**Table 2:** Classification features for six entity types

Fields of page	Classification features for six entity types
Entity name	Ends with any words in (department, disease, inflammation, tumour, syndrome, examination)
Abstract	Contains any words in (symptom, syndrome, symptoms of illness, disease name of TCM)
Content	Has more than 3 words in (function, specification, adverse reaction, side effect, component, usage, dosage),
	Has more than 3 words in (cause, examination, antidiastole, diagnosis, mitigation, pathogenesis, clinical manifestation)
Full-text	Contains any words in (Chinese patent medicine, Chinese herbal medicine)
Category	Contains any words in (medicine, disease, TCM, drug, Chinese patent medicine, symptom)

#### Data extraction from EMRs

Due to variations of symptoms in clinical practice, we incorporate clinical vocabularies into our KB by extracting symptoms from a large number of EMRs. In order to learn symptoms of Western medicine, we extract texts from the “*Physical* *Examination*” and “ *Antidiastole_Western* *medicine*” fields of EMRs. Texts within “*Disease* *Analysis*” and “*Antidiastole* *TCM*” fields are selected for TCM symptoms. Since data duplication takes place frequently in the texts of EMRs, we remove the sentences appearing more than once in the same record. We manually annotate TCM symptoms and symptoms of Western medicine in EMRs. Then we train a CRF [20] classifier to recognize new symptoms. The features are classified as literal features, position features, and part-of-speech (POS) features as shown in Table 3. The literal features and position features are adopted from [21]. We set the context window size to 3 since it achieves best results in [21].

**Table 3:** Features of CRF

Feature type	Feature contents	
Literal features	Unigram	*X*_*i*−3_, *X*_*i*−2_, *X*_*i*−1_,*X*_*i*_, *X*_*i*+1_, *X*_*i*+2_, *X*_*i*+3_
	Bigram	*X*_*i*−3_ *X*_*i*−2_,*X*_*i*−2_ *X*_*i*−1_,*X*_*i*−1_ *X*_*i*_, *X*_*i*_ *X*_*i*+1_, *X*_*i*+1_ *X*_*i*+2_, *X*_*i*+2_ *X*_*i*+3_
	Trigram	*X*_*i*−3_ *X*_*i*−2_ *X*_*i*−1_,*X*_*i*−2_ *X*_*i*−1_ *X*_*i*_, *X*_*i*−1_ *X*_*i*_ *X*_*i*+1_,*X*_*i*_ *X*_*i*+1_ *X*_*i*+2_, *X*_*i*+1_ *X*_*i*+2_ *X*_*i*+3_
Position features	Index _*i*_	
POS features	Unigram	*P*_*i*−3_, *P*_*i*−2_, *P*_*i*−1_, *P*_*i*_, *P*_*i*+1_, *P*_*i*+2_, *P*_*i*+3_
	Bigram	*P*_*i*−3_ *P*_*i*−2_, *P*_*i*−2_ *P*_*i*−1_,*P*_*i*−1_ *P*_*i*_, *P*_*i*_ *P*_*i*+1_, *P*_*i*+1_ *P*_*i*+2_, *P*_*i*+2_ *P*_*i*+3_
	Trigram	*P*_*i*−3_ *P*_*i*−2_ *P*_*i*−1_,*P*_*i*−2_ *P*_*i*−1_ *P*_*i*_, *P*_*i*−1_ *P*_*i*_ *P*_*i*+1_,*P*_*i*_ *P*_*i*+1_ *P*_*i*+2_, *P*_*i*+1_ *P*_*i*+2_ *P*_*i*+3_

### Data fusion

Data fusion consists of three steps: entity type alignment, entity mapping, and attribute mapping.

Firstly, We align entity types with a voting method, i.e., the entity type receiving the most votes wins. When two types have the same top votes,the entity type with the higher priority wins. Priorities are determined by the resources’ rankings in Alexa.

Secondly, we use the idea of Wang et al. [22] to map entities. They used two variables, namely, *commonness* and *relatedness* in combination to calculate similarities between entities. In this paper, the *commonness* is obtained by calculating string similarities between entity names, while the *relatedness* is calculated with string similarities of attribute values. For example, entity *E*_*A*_ and *E*_*B*_ have the same type and share two attributes *A*_1_ and *A*_2_. The *commonness* is defined as

1 $$ StringSimilarity(E_{A},E_{B})= \frac{\left|LCS(E_{A},E_{B})\right|}{Max(\left|E_{A}\right|,\left|E_{B}\right|)}  $$

where |*LCS*(*E*_*A*_,*E*_*B*_)| is the length of the longest common subsequence between *E*_*A*_ and *E*_*B*_. The *relatedness* is the ratio of the number of similar facts. If the product of *commonness* and *relatedness* is higher than a threshold, we will map *E*_*A*_ to *E*_*B*_.

Finally, we map attribute from the extraction results to our ontology. Since property boxes of each healthcare website share the same attribute names, we manually map attribute names in healthcare websites to our ontology. However, in Chinese encyclopedia sites, the infoboxes of entity pages exist lots of attribute names that are similar in semantics but different in names. We map attributes to our ontology according to type information of entities and attribute values. For example, attribute “symptom” of triple <vertigo of heat stroke, symptom, thirsty > is mapped into “symptom related symptom”, because entity “vertigo of heat stroke” and attribute value “ thirsty” are both symptoms.

### Link to UMLS

To investigate similarities and differences between symptoms in Chinese and English, we link our KB to UMLS, a medical KB widely used in clinical practice and medical informatics research. Xu et al. [23] used context similarities to link phrases in “English discharge summary” to “Chinese discharge summary”. However, there are no such contexts in our situation. We first call the API of Baidu Translate [24] to translate symptoms in our KB into English phrases. Second, each phrase is transformed into a bag of words (denote as *BW*_*CS*_), and the same operation is done to concepts in UMLS (denote as *BW*_*UMLS*_). Third, the *JaccardSimilarity* [25] between elements in *BW*_*CS*_ and *BW*_*UMLS*_ is calculated. Only when the value of *JaccardSimilarity* is 1, do we make a linkage.

## Results and discussion

### Classification results

**Fig. 4:**
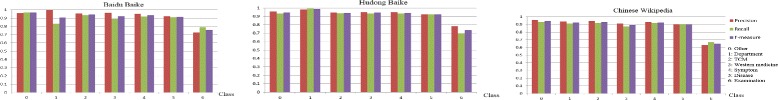
Classification Results for Encyclopedia Sites. The result for Examination is obviously lower than those for the other classifications, because some entity pages in encyclopedia sites are irregular, leading to few features being used. Besides, Chinese Wikipedia has a few seed entities of examination, so its result is worse than those for other encyclopedias

We apply Decision Tree algorithm to train a multi-class classifier. Twenty-six features from five fields of entity pages in encyclopedia sites are utilized in this paper. We use ten fold cross-validation. Figure 4 shows the results of our classifier in three encyclopedia sites, and indicates that our classifier has high accuracy and recall.

### EMR data extraction results

We have collected 250,000 EMRs from Shanghai Shuguang Hospital as our corpora. Two TCM experts annotate symptoms mentioned in 1000 EMRs, which are randomly divided into two parts. One containing 660 EMRs form the training set, and the rest form the test set. Then we train a CRF model. The precision of TCM symptom is 93.26%, and the precision of symptom of Western medicine is 95.37%. Finally, we use the model to recognize new symptoms from 249,000 EMRs. We have extracted 2376 symptoms, 387 of which are TCM symptoms and 1989 of which are symptom of Western medicine.

### Statistic

**Fig. 5:**
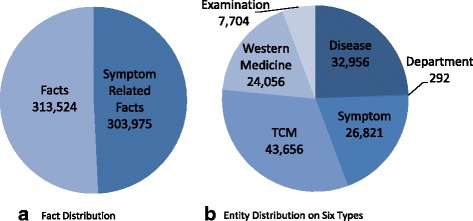
Data Distribution on our KB. From (**a**), symptom-related facts account for 49.23% in all facts of our KB, which is the result of symptoms being the focus of our KB. From (**b**), we find that medicine entities account for 50% of all entities, and 64.4% of them are TCM

Our KB has 135,485 distinct entities and 617,499 facts. More precisely, there are 26,821 symptoms, 32,956 diseases, 67,712 medicines, 292 departments, and 7704 examinations, shown in Fig. 5. Table 4 shows the distribution of entities from each source and the ultimate results of our KB. We find: (1) The number of symptoms from all sources is 41,020. It is far larger than the number of distinct symptoms (i.e. 26,821) in our KB, which shows the large-scale duplication between different data sources. (2) The website that contributes most to symptoms in our KB is *fh*21. It contains 9780 symptoms and accounts for only 36.5% of our KB, which shows the advantage of collecting data from different sources. (3) The EMRs also add 2376 new symptoms to our KB, showing the differences between symptoms used in websites and in clinical practice.

**Table 4:** Entity evaluation of different data sources

Entity Type	Precision	Harvested entities												
		Family doctor	JIANKE	120ask	QQYY	39Health	99Health	fh21	PCbaby	Baidu Baike	Hudong Baike	Chinese Wikipedia	EMRs	Result
Symptom	0.981	4775	7748	7610	4123	6659	5745	9780	1787	2932	997	393	2376	26821
Disease	0.967	9998	8004	7138	2546	7778	344	3991	1389	5688	4184	587	–	32956
Medicine	0.983	893	1281	3271	2175	6325	1423	5121	879	22152	27469	365	–	67712
Department	0.976	31	–	55	12	37	31	–	–	192	125	53	–	292
Examination	0.783	–	1403	302	–	2909	–	–	–	230	301	39	–	7704
Aggregated^a^	0.938	15697	18436	18376	8856	23708	7543	18892	4055	31194	33076	1437	2376	135485

^a^Precision values are averaged and numbers of harvested entities are summed

For correctness evaluation, we use “*correctness* *ratio* *of* *facts*” [26] to evaluate symptom-related facts. Each sampled triple is evaluated by seven persons according to their knowledge. We integrate the evaluating results by voting. Since our KB is large, we use simple random sampling method to draw samples.

Based on [26], we sampled 417 triples from 26,821 rdf:type triples whose objects are Symptom to calculate the correctness of symptoms. Its *correctness* *ratio* is 98.1%. Then, we sampled 423 triples from 295,946 symptom related triples. The *correctness* *ratio* of symptom-related facts is 95.9%.

**Fig. 6:**
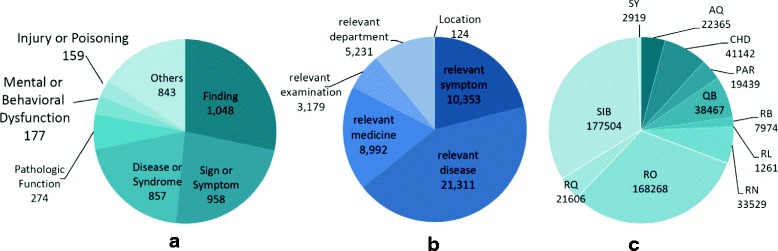
Data Distribution on the Linked Results. **a** Semantic Type Distribution on Linked Concepts in UMLS, **b** Property Distribution on Linked Symptom in our KB, **c** Property Distribution on Linked Concepts in UMLS

We have collected 4298 links between symptoms in our KB and concepts in UMLS (abbreviate as symptom links). We sampled 385 triples to evaluate the correctness of the links, and the *correctness* *ratio* is 92.0%. We calculate the semantic type distribution on the symptom links in UMLS, shown in Fig. 6 a. Normally, symptoms in our KB are expected to link to concepts in Sign or Symptom or Finding. But 53.3% of symptoms are linked to other semantic types in UMLS. For example, “Icterus (C0022346)” and “infectious jaundice (C0241954)” are common symptoms in Chinese, while the semantic type of the former one is “Pathologic Function” and the latter one is Disease or Syndrome. This shows the range of symptom in life is much broader than that in medical science. Attribute distribution on symptom links in the two KBs are shown in Fig. 6 b and c. We define six attributes for symptoms in our KB, and UMLS defines 13 attributes for the linked concepts. However, most properties in UMLS do not have fixed domains and ranges. Thus, these attributes can not be interpreted uniformly. For example, “RO” in UMLS refers to an uncertain relation. In contrast, our KB provides relations with definite syntax and semantics.

## Conclusions and future work

The KB is constructed by fusing data automatically extracted from eight mainstream healthcare websites, three Chinese encyclopedia sites, and symptoms extracted from a lager number of EMRs as supplement. Finally, we obtain 135,484 entities to our KB, among them, the number of symptom entities is 26,821. The KB can be used to annotate symptoms in EMRs. It can also be embedded into EMR systems to help therapists with symptom recommendations. In the future, we will try to construct a whole KB of commonly used medical vocabularies in Chinese, linking them to UMLS concepts.

## Abbreviations

CRF: Conditional random field
EMR: Electronic medical record
KB: Knowledge base
POS: Part-of-speech
TCM: Traditional Chinese medicine
UMLS: Unified medical language system
